# Supplementary material for: Low-frequency conductivity of low wear high-entropy alloys
Source: Nat Commun. 2024 May 29;15:4554. doi: 10.1038/s41467-024-49035-0 (PMC11136967; doi:10.1038/s41467-024-49035-0)
Supplement: Supplementary file 1 — Supplementary Information [file 41467_2024_49035_MOESM1_ESM.pdf]

Supplementary Information

Low-frequency conductivity of low wear high-entropy alloys

Cheng-Hsien Yeh [1], Wen-Dung Hsu [2,3,4] *, Bernard Haochih Liu [2,3] *, Chan-Shan Yang [3,5] *, Chen-Yun Kuan [2], Yuan-Chun Chang [2], Kai-Sheng Huang [2], Song-Syun Jhang [2], Chia-Yen Lu [5], Peter K. Liaw [6], and Chuan-Feng Shih [1,3,4] *

[1] Department of Electrical Engineering, National Cheng Kung University, Tainan 70101, Taiwan

[2] Department of Materials Science and Engineering, National Cheng Kung University, Tainan 70101, Taiwan

[3] Applied High Entropy Technology (AHET) Center, National Cheng Kung University, Tainan 70101, Taiwan

[4] Program on Semiconductor Packaging and Testing, Academy of Innovative Semiconductor and Sustainable Manufacture, National Cheng Kung University, Tainan 70101, Taiwan

[5] Institute and Undergraduate Program of Electro-Optical Engineering, National Taiwan Normal University, Taipei 11677, Taiwan

[6] Department of Materials Science and Engineering, The University of Tennessee, Knoxville, TN 37996, USA

*E-mail: cfshih@mail.ncku.edu.tw (Chuan-Feng Shih), wendung@mail.ncku.edu.tw (Wen-Dung Hsu), hcliu@mail.ncku.edu.tw (Bernard Haochih Liu), csyang@ntnu.edu.tw (Chan-Shan Yang)

Supplementary Note 1 | Calculation of frequency-dependent conductivity

The mathematical representation of the Drude-Lorentz model is as follows:

$$\sigma_{DL}(\omega) = \sum \frac{\varepsilon_0 \omega_{Dp}^2 \tau_D}{1 - i\omega\tau_D} + \sum \frac{i\varepsilon_0 \omega_{Lp}^2 \omega}{-\omega^2 - i\omega\gamma_L + \omega_L^2} \tag{1}$$

The right-hand side of the equation consists of two terms: the first term represents the Drude model, and the second term represents the Lorentz model, where ε_0 is the vacuum permittivity, ω is the frequency of the applied external electric field, ω_{Dp} is the electron plasma frequency in the Drude model. τ_D is the electron relaxation time in the Drude model. ω_{Lp} is the electron plasma frequency in the Lorentz model, γ_L is the reciprocal of the electron relaxation time in the Lorentz model, and ω_L is the electron-resonance frequency in the Lorentz model.

The Lorentz model describes electrons that are bound by atomic nuclei and behaves like a mass-spring system. Consequently, this system exhibits a resonance frequency. It is important to note that the resonance frequency in the Lorentz model and the plasma frequency arise from different physical phenomena. The former represents the natural resonance frequency of electrons paired with atomic nuclei, while the latter denotes the oscillation frequency of electrons interacting with other surrounding electrons.

Furthermore, within a material, there may be both free and bound electrons affected by various interactions. Hence, there can be different values for ω_{Dp}, τ_D, ω_{Lp}, γ_L, and ω_L. Therefore, to describe the overall frequency-dependent conductivity of a material, a sum of the multiple Drude and Lorentz model terms is used, as explained in Equation (1). If consider the dc-conductivity(σ_0), Equation (2) can be obtained by setting ω to 0 in Equation (1).

$$\sigma_0 = \sum \varepsilon_0 \omega_{Dp}^2 \tau_D \tag{2}$$

From Equation (2), it can be inferred that the primary factors influencing the DC conductivity are ω_{Dp} and τ_D.

The ω_{Dp} in the Drude model can be likened to the oscillation frequency of the perturbed electron cloud. Its value is correlated with the viscosity of the electron cloud. This phenomenon can be analogized to viewing the electron cloud as a fluid (with viscosity, where this viscosity is related to the density of the fluid and the mass of the molecules constituting it). If an external force imparts a disturbance in the fluid, waves will be generated, akin to the oscillation observed in a container of water. The frequency of these oscillations can be analogously compared to the plasma frequency. This frequency can be estimated, using Equation (3).

$$\omega_{Dp}^2 = \frac{ne^2}{\varepsilon_0 m_0} \tag{3}$$

In this equation, n represents the density of free electrons, m_0 denotes the effective mass of free electrons, and *e* stands for the charge of a free electron. The relaxation time (τ_D) refers to the average time interval between collision events that occur when free electrons are in motion. These collisions can take the form of electron-electron interactions, electron-nucleus interactions, or electron-defect interactions. This time interval is intimately related to the material's microscopic structure, such as whether the lattice is subject to stress-induced strain or if defects are present. The relaxation time can be estimated, employing Equation (4),

$$\tau_D = \frac{m_0 \sigma_0}{ne^2} \tag{4}$$

where σ_0 represents the DC conductivity.

In calculations to estimate low-frequency conductivity, according to the Drude model [Equation (2)], it is necessary to know ω_{Dp} and τ_D. The plasma frequency (ω_{Dp}) can be calculated from first principles, while estimating the relaxation time is the most challenging part of calculating low-frequency conductivity. This is because, as mentioned earlier, this value is fundamentally related to the microstructure (which affects the probability of electron collisions during motion) and is also a function of temperature. Currently, there is no reliable theoretical derivation available for estimation. Predicting the low-frequency conductivity of HEAs is even more difficult because most HEAs have a multiphase solid-solution

microstructure, with multiple components randomly occupying lattice positions. While the lattice structure of this material remains ordered, the composition is disordered. This compositional disorder leads to random lattice distortions, thereby affecting the estimation of the relaxation time.

In this work, the relaxation time is measured, using THz Time-domain Spectroscopy, and the detailed method is given in Supplementary Note 3 and Supplementary Figs. 4 and 5.

In general, the various properties of HEAs can be estimated by a linear combination of their compositions. In HEAs. However, this approach overlooks the fact that the bonding interactions between atoms in HEAs, when different coordinating elements are present. This random variation in coordinating elements can cause deviations in the electronic density of states of HEAs from what would be expected, based on a linear combination of pure elemental compositions. Consequently, the plasma frequency (ω_{Dp}) deviates from a linear combination as well. Additionally, the random lattice distortions in the solid-solution microstructure of HEAs can also cause deviations in the relaxation time from a linear combination.

Supplementary Note 2 | Simulation results of frequency-dependent conductivity, plasma frequency, and free-electron density

The experimental DC conductivity, plasma frequency, and relaxation time values of pure elements are listed in Supplementary Table 1. The three estimation methods of conductivities are summarized in Supplementary Table 2. To understand how the conductivity deviates from linear combinations when forming HEAs, it is essential to investigate how the plasma frequency (ω_{Dp}) and relaxation time (τ_D) change. We can compare the differences in ω_{Dp} calculated from linear combinations of pure elements with that obtained from the high-entropy-alloy model. Additionally, we can estimate the changes in the free-electron density and effective electron mass through the calculation methods to explain the variations in ω_{Dp}.

Regarding the relaxation time, we can compare the lattice constants and root mean square differences in atomic positions within the unit cell of HEAs with those derived from linear combinations of pure elements. This comparison will help us understand how lattice distortions impact relaxation time. By comparing these parameters between HEAs and linear combinations of pure elements, we can gain insights into how the formation of HEAs affects their conductivity and how deviations from ideal linear behavior occur. From the plasma frequencies for the various elements, we can calculate the plasma frequencies according to linear combinations in the order of Mo plus W comprising 70 mol%, equimolar proportions, and Ta plus W comprising 70 mol% to be 8.62 eV, 8.73 eV, and 8.59 eV, respectively. In comparison, the plasma frequencies directly calculated from the HEA model are significantly lower, being 4.66 eV, 4.54 eV, and 3.77 eV, respectively, listed in Supplementary Table 3. This substantial difference indicates that the atomic bonding between different elements during the formation of HEAs has a significant impact on the plasma frequency. According to Equation (3), the primary factors influencing the plasma frequency are the free-electron density and the effective electron mass.

By calculating the density of states and using the Fermi-Dirac distribution, we can estimate the free-electron density at room temperature (300 K). Supplementary Table 3 presents the calculated results of free-electron densities. It is evident that the free-electron density for the pure-elemental models is on the order of 10^{28} electrons/m^3 at room temperature, while after forming HEAs, the free electron density drops to the order of 10^{26} electrons/m^3. This result indicates that when HEAs are formed, the bonding interactions between different metallic elements significantly reduce the free-electron density. This reduction in the free-electron density is attributed to the substantial differences in the electronegativity between the metallic elements. Compared to the linear combination, the free-electron density is approximately only 0.014 - 0.02 times that of the linear combination.

When comparing the free-electron densities among the three NbMoTaW films, it can be observed that the equimolar model as the highest free-electron density, the Ta plus W comprising 70 mol% model has the second-highest free-electron density, which is close to the equimolar model, and the Mo plus W comprising 70 mol% model has the lowest free-electron density. This trend in the free-electron density is similar to that observed in plasma frequencies. However, it is worth noting that the order of the equimolar model and the Ta plus W comprising 70 mol% model is reversed when compared to the plasma-frequency trend.

Next, we examine the impact of the effective-electron mass. The effective-electron mass can be calculated from electronic-band structures. Nb, Mo, Ta, W, and the NbMoTaW system all have a BCC structure. When calculating band structures, a primitive cell and the corresponding reciprocal space K-point path (K-path) need to be defined. The K-path is typically set to describe the electronic states fully and follows the high-symmetry points in the first Brillouin zone of the BCC structure ($\Gamma{-}H{-}N{-}\Gamma{-}P{-}H|P{-}N$). Supplementary Figs. 1 and 2 shows the calculated band structures.

When calculating the effective mass of electrons in semiconductors or insulators, Equation (5) is used to perform a second derivative of the valence-band maximum and

conduction-band minimum in the band structure. This derivative provides information about the effective mass of electrons in the material.

$$\frac{1}{m_0} = \frac{1}{\hbar}\frac{\partial^2 E}{\partial^2 k} \tag{5}$$

where m_0 represents the effective-electron mass, $\hbar$ is the reduced Planck constant, E is the electron energy, and k is the reciprocal space path. However, the materials are conductors, and the energy bands directly cross the Fermi level. Hence, it is challenging to distinguish between valence and conduction bands on the electron-band structure diagram. Therefore, the concept of an effective mass is utilized to express the ability of electrons near the Fermi energy to respond to an applied electric field. In this calculation, because there are several bands crossing the Fermi energy, an average value is taken. The calculated results are shown in Supplementary Table 3, indicating that the effective electron mass for the pure elemental Nb, Mo, Ta, and W is approximately in the range of 0.85 - 2.86 times the electron mass. However, after forming HEAs, the effective electron mass increases to the range of 4.61 - 6.26 times the electron mass, which is approximately four times than the linear combination value. This feature suggests that after forming HEAs, a larger electric field is required to achieve the same electron mobility. Compared to the effective-electron mass of the linear combination, these values are approximately 3.34 - 4.00 times larger, indicating a significant increase in the electron mass when HEAs are formed.

Next, we observe the correlation between the lattice distortion and relaxation time. To quantify the extent of lattice distortion, our discussion is divided into two parts. That is, the sign of the percentage deviation of lattice constants from linear combinations is positive for expansion and negative for contraction, listed in Supplementary Table 4. The root-mean-square deviation of atomic positions from ideal locations within the lattice, also listed in Supplementary Table 4.

When comparing the relaxation times of pure elements with those of HEAs, it is evident that both relaxation times are in the same order of magnitude, approximately 10^{15} seconds. However, the relaxation times of HEAs are shorter than those of pure elements, and they also deviate from the values predicted by linear combinations based on elemental proportions. Lattice distortion can be attributed as the main cause of this deviation.

Supplementary Table 4 presents the percentage deviation of lattice constants from linear combinations and the root mean square deviation of atomic positions within the lattice for the three proportions of NbMoTaW HEAs. It is observed that all three compositions show deviations from ideal values in both lattice constants and atomic positions, although these deviations are very small, with lattice constants on the order of 10^{-4} Å and even smaller differences in atomic positions. Despite the small magnitude of these deviations, they still have some impact on relaxation time. However, it is important to note that the relaxation times listed in Supplementary Table 4 are determined through experimental measurements, which may introduce some uncertainty. What can be confirmed is that the formation of HEAs results in a reduction in the electron-relaxation time, approximately one-fifth of the linear-combination value in the case of this study. The calculated lattice constants are listed in Supplementary Table 5.

Therefore, based on the analysis above, it is inferred that the reduction in the conductivity of HEAs is due to the decrease in the plasma frequency and the decrease in the relaxation time. These results explain the deviation of conductivity obtained directly from the cocktail effect. In the case of the NbMoTaW series studied in the present research, the plasma frequency is approximately half of its linear combination value, while the relaxation time is one-fifth, resulting in an overall conductivity that is one-tenth of the linear combination value. To improve the precision of this analysis to the point where it can differentiate between conductivity differences in different composition ratios, a more accurate theoretical approach for estimating the relaxation time would be required.

Using equation (6), the dielectric equation can be derived, as shown in Supplementary Fig. 3. Substituting the fitting parameters into Equation (1) yields the frequency-dependent conductivity, where ε_∞ is the dielectric constant at an infinite frequency, the ideal electron gas being 1, ω_{Dp} and ω_{Dp} are the plasma frequencies for the Drude and Lorentz models, γ_d is the electron collision frequency, γ_L is the damping coefficient, and ω_L is the resonant frequency.

$$\varepsilon_{DL}(\omega) = \varepsilon_\infty - \sum \frac{\omega_{Dp}^2}{\omega^2 + i\omega\gamma_d} - \sum \frac{\omega_{Lp}^2}{\omega^2 - i\omega\gamma_L - \omega_L^2} \quad (6)$$

Supplementary Note 3 | Measurement of Carrier relaxation Times Based on THz Time-domain Spectroscopy

Terahertz (THz) time-domain spectroscopy (THz-TDS) has been demonstrated as a powerful tool for measuring the complex electrical properties, such as scattering time, plasma frequencies, carrier densities, and mobilities, of charge carriers in semiconductors by analyzing the time-dependent response THz pulses [1-6]. A schematic of the transmission-type THz-TDS is shown in Supplementary Fig. 4.

The basic principle of the THz TDS is to generate and detect THz pulses using an ultrafast laser technology. Here, we employed a seed-laser device (Vitara–Flexible Ultrashort Pulse Ti: Sappire Laser) that produced a laser beam with a wavelength of 532 nm and a repetition rate of 80 MHz. The laser beam passed through an amplifier (Legend Elite HE+ USP-5K-III) and thus gained a center wavelength of 800 nm, an average output power of 8.2 W, a pulse width smaller than 35 fs, and a repetition rate of 5 KHz. The amplified beam was then split by a beam splitter into two beams; one is termed as the "pump beam" (for producing THz signals), and another, the "probe beam" (for probing THz radiation). Then, we directed the pump beam to pass through a chopper to raise the signal-to-noise ratio (SNR). Finally, the pump beam was directed to pass through a nonlinear crystal—ZnTe—to generate the THz radiation by the beat frequency. Additionally, a silicon substrate with a high electrical resistivity was placed behind the ZnTe crystal to filter the fundamental beam so that only THz radiation could pass through the substrate. A pair of parabolic mirrors was used to calibrate the THz radiation and focus it onto a sample. Another pair of parabolic mirrors was employed to collect the THz radiation that passed through the sample and focus it onto the ZnTe crystal at the probing end to conduct the measurement by electro-optic sampling.

Considering the Maxwell equations and a simple conducting medium, the equivalent complex dielectric function consisting of contributions from conduction-band electrons and bound electrons can be demonstrated.

Further, through the relation between the dielectric function and conductivities, we can determine the complex THz conductivity in the HEAs thin films, $Nb_{25}Mo_{25}Ta_{25}W_{25}$, $Nb_{15}Mo_{15}Ta_{35}W_{35}$, and $Nb_{15}Mo_{35}Ta_{15}W_{35}$, respectively, the Drude-Smith model , a kind of non-Drude behavior consisting of contributions from conduction-band and bound electrons. Complex THz conductivities ($\sigma^* = \sigma_{Re} + i\ \sigma_{Im}$) are plotted as a function of frequency in Supplementary Figs. 5a and 5b. Supplementary Table 2 summarizes the scattering times, τ, of $Nb_{25}Mo_{25}Ta_{25}W_{25}$, $Nb_{15}Mo_{15}Ta_{35}W_{35}$, and $Nb_{15}Mo_{35}Ta_{15}W_{35}$, respectively. The order of their scattering times is always around the level of femtosecond.

Supplementary Note 4 | The estimation of optimal concentration of Nb-Mo-Ta-W system with good mechanical properties and conductivity

To validate this assertion across diverse mechanical properties—encompassing C_{11}, C_{12}, C_{44}, bulk modulus, shear modulus, and Young's modulus—of the three NbMoTaW HEA compositions under discussion, we have plotted them based on the first principles calculated results of the HEA models and the corresponding properties weighted by composition, as shown in Supplementary Table 9 and Fig. 19. Across various mechanical properties encompassing Nb, Mo, Ta, and W, the hierarchy of superiority follows W > Mo > Ta > Nb, a trend echoed in conductivity as well. Therefore, to optimize the mechanical properties and conductivity of NbMoTaW HEAs, augmenting the proportions of W and Mo yields the most favorable results. Nonetheless, fluctuations in composition also impact the stability of the high-entropy solid solution phase. Consulting the phase diagrams generated by Pandat[7], the transformation temperatures from the HEA phase to the B2 phase are plotted in Supplementary Fig. 20 based on the composition ratios of Nb, Mo, and W. Generally, the B2 phase exhibits stability at lower temperatures, whereas the HEA phase is stable at higher temperatures. Lower transformation temperatures suggest that the HEA phase can exist more readily under ambient conditions. The figure suggests that higher proportions of Mo and W lead to lower transformation temperatures. However, if the composition ratio exceeds 35 mol%, there needs to be at least 0.15 mol% of Nb present to ensure that the HEA phase transformation temperature remains below 400°C.

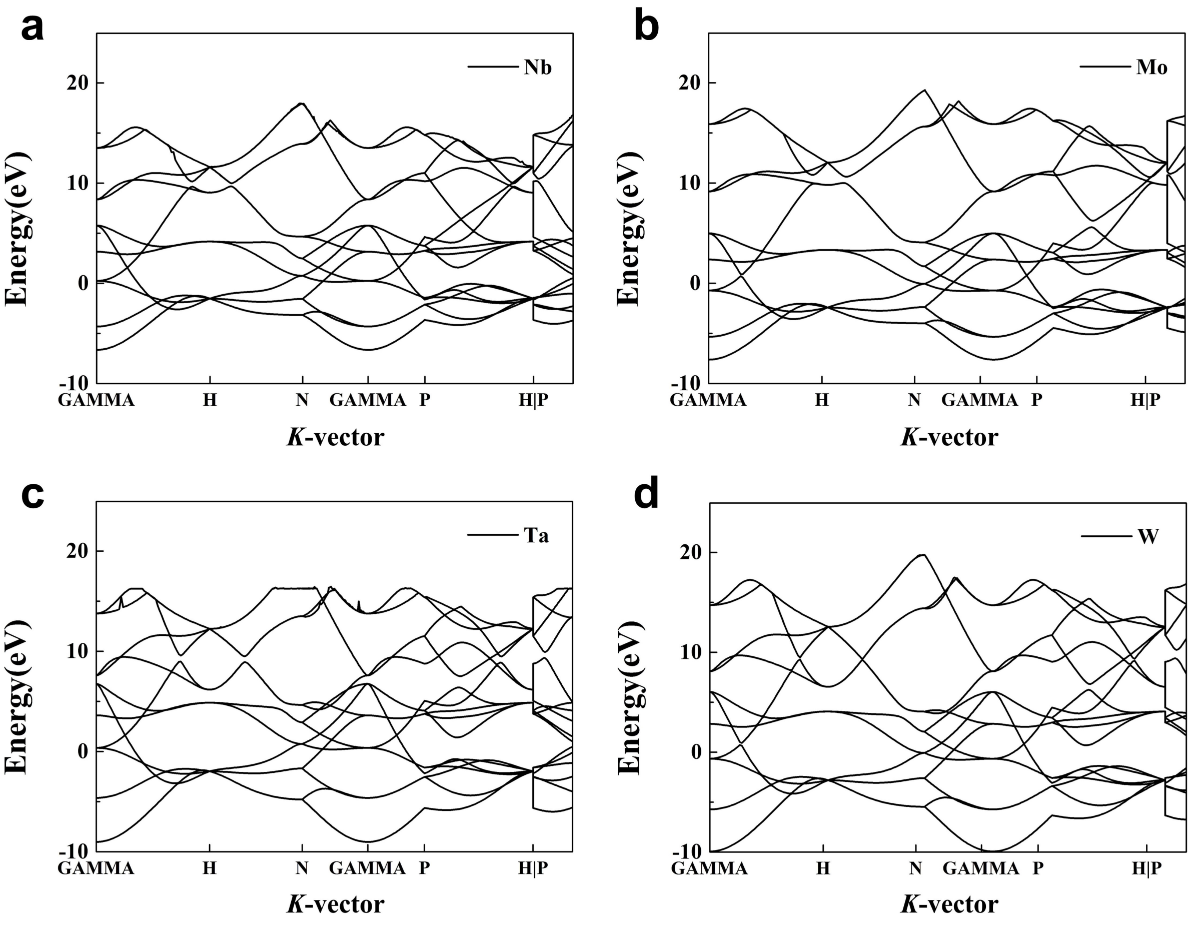

Supplementary Fig. 1 Band structure of elements. **a** Nb. **b** Mo. **c** Ta. **d** W.

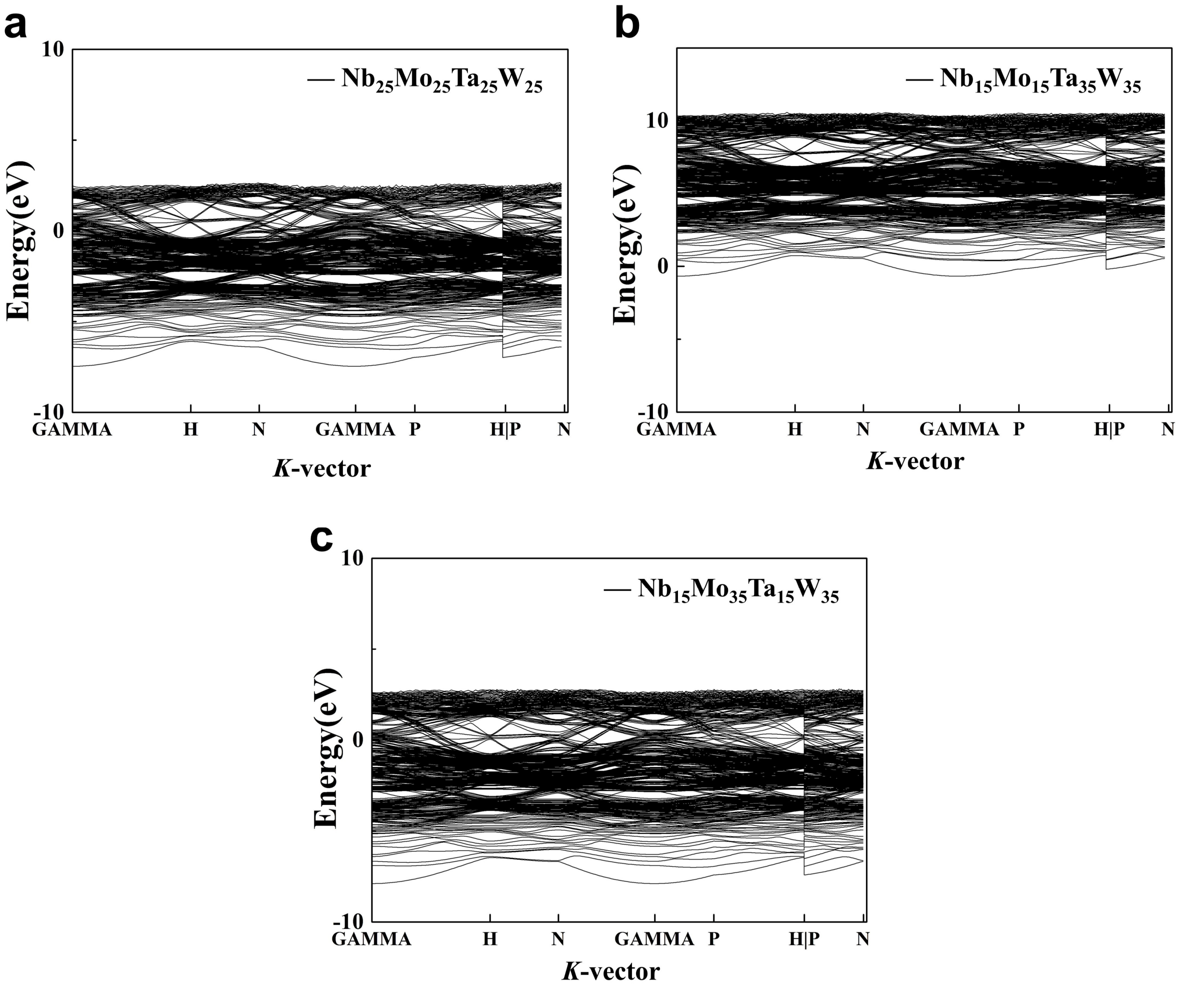

Supplementary Fig. 2 Band structure of Nb-Mo-Ta-W high entropy alloy. **a** $Nb_{25}Mo_{25}Ta_{25}W_{25}$. **b** $Nb_{15}Mo_{15}Ta_{35}W_{35}$. **c** $Nb_{15}Mo_{35}Ta_{15}W_{35}$.

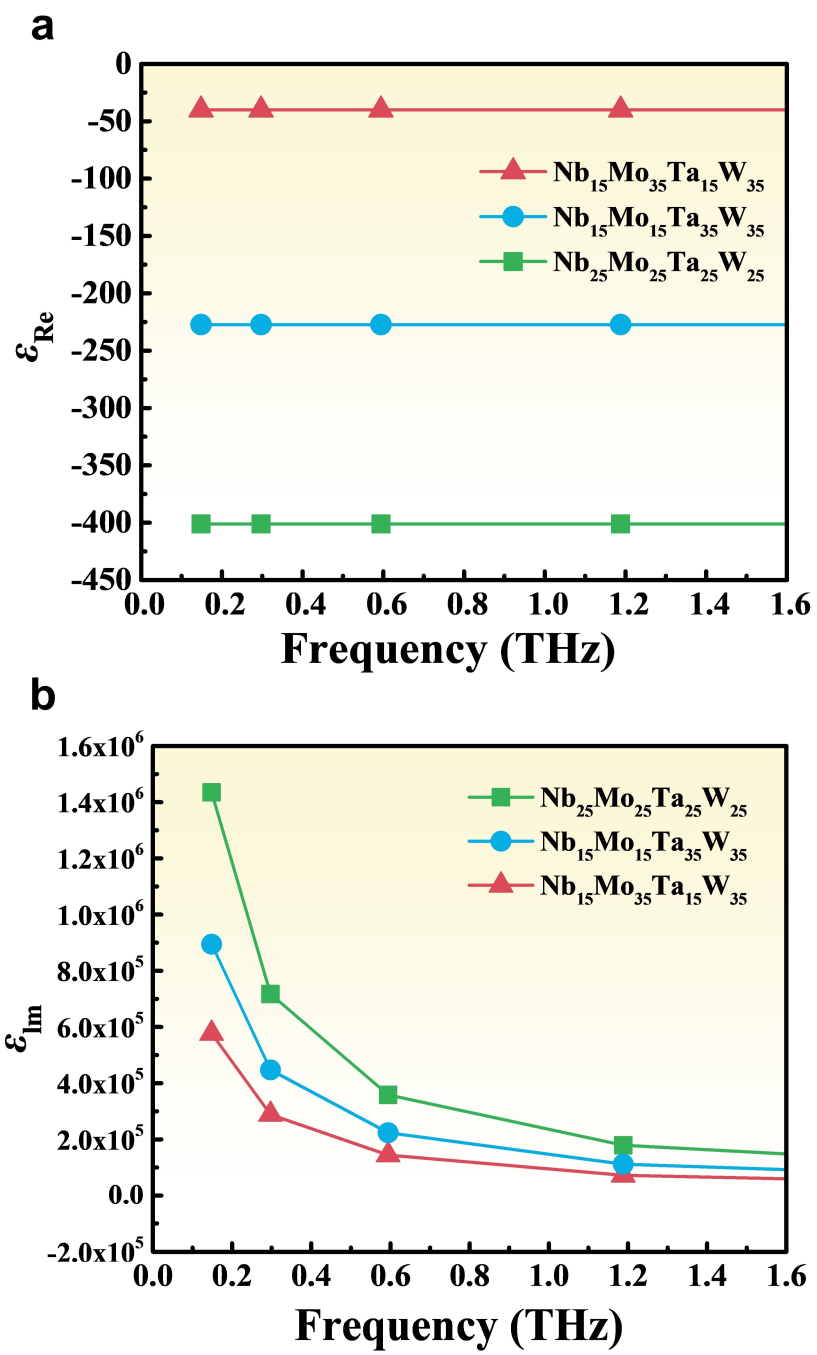

Supplementary Fig. 3 The **a** real and **b** imaginary parts of the dielectric constants in the NbMoTaW alloy system.

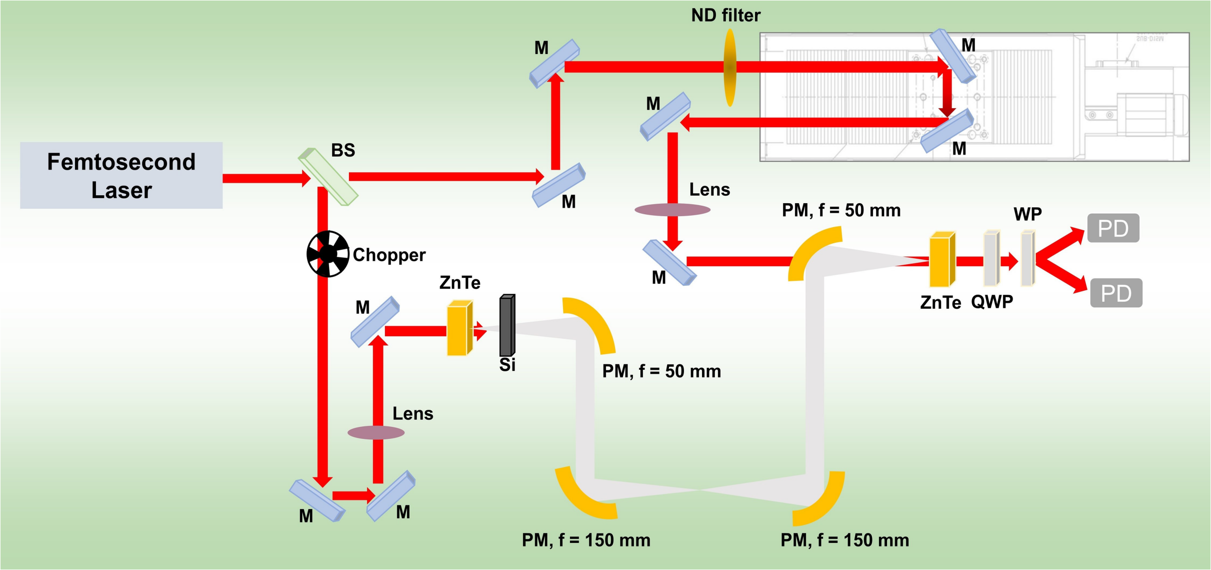

Supplementary Fig. 4 The schematic of transmission-type THz time-domain spectroscopy. (M: mirror. BS: Beam Splitter. HWP: Half Wave Plates. PM: off Axis Parabolic mirror. QWP: Quarter Wave Plates. WP: Wollaston Prism. PD: Photodetector.)

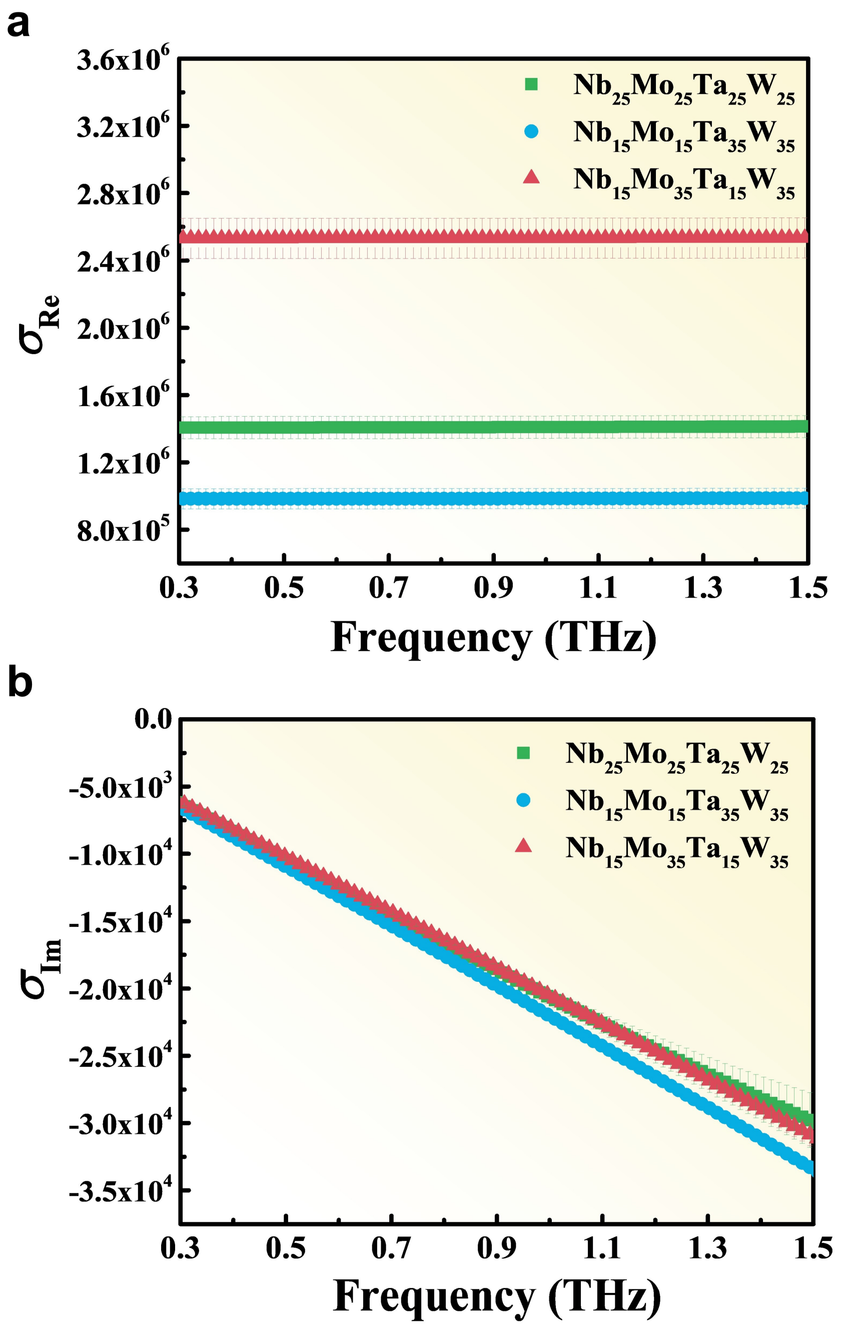

Supplementary Fig. 5 The **a** real and **b** imaginary part of complex conductivities of HEAs thin films (~10 nm), $Nb_{25}Mo_{25}Ta_{25}W_{25}$, $Nb_{15}Mo_{15}Ta_{35}W_{35}$, and $Nb_{15}Mo_{35}Ta_{15}W_{35}$. The error bars represent the standard deviation.

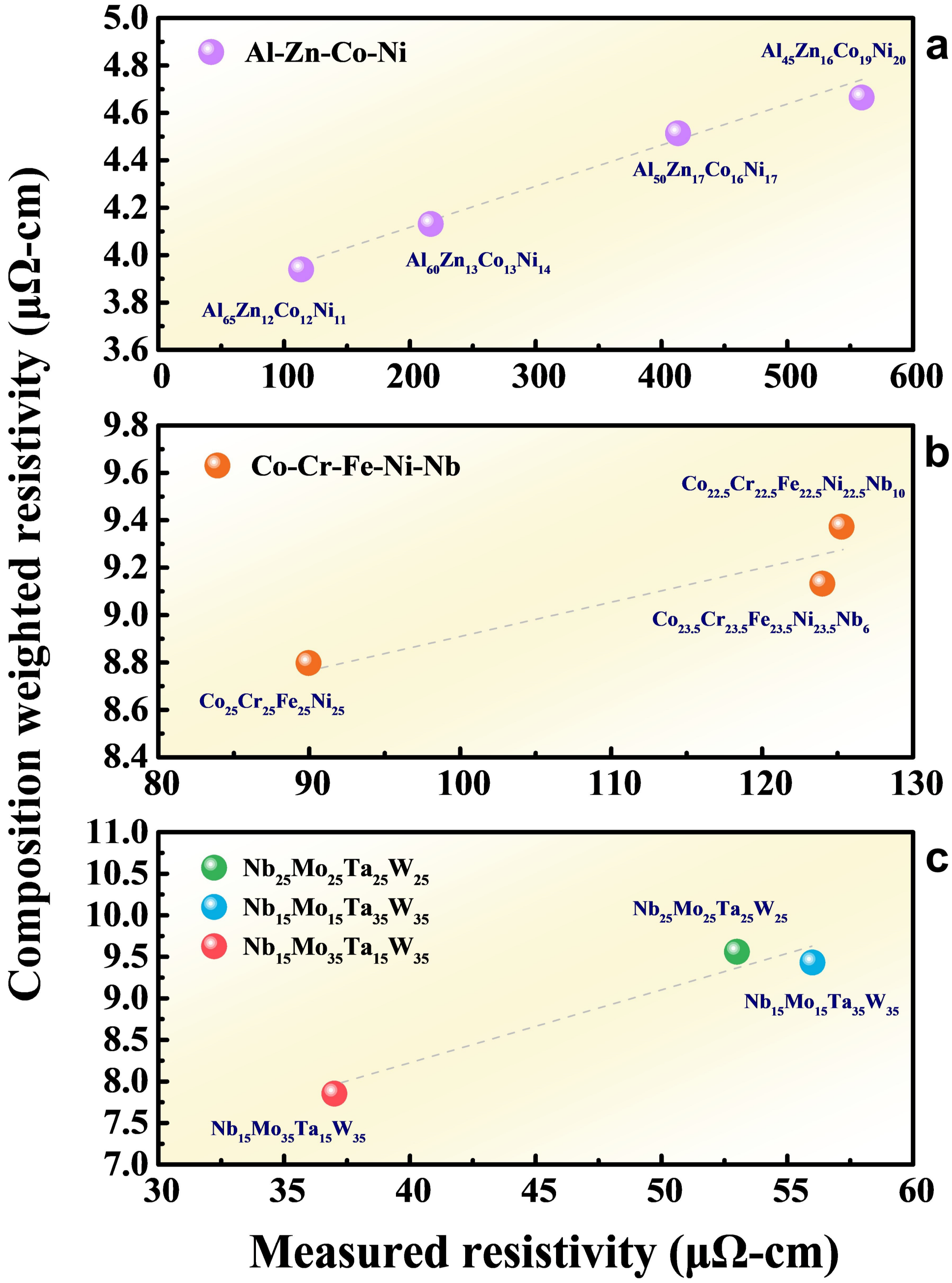

Supplementary Fig. 6 Linear combination of resistivity versus measured resistivity of literature and this work. **a** Al-Zn-Co-Ni films[8]. **b** Co-Cr-Fe-Ni-Nb high entropy alloy films[9]. **c** This work with $Nb_{25}Mo_{25}Ta_{25}W_{25}$, $Nb_{15}Mo_{15}Ta_{35}W_{35}$, and $Nb_{15}Mo_{35}Ta_{15}W_{35}$ high entropy alloy films.

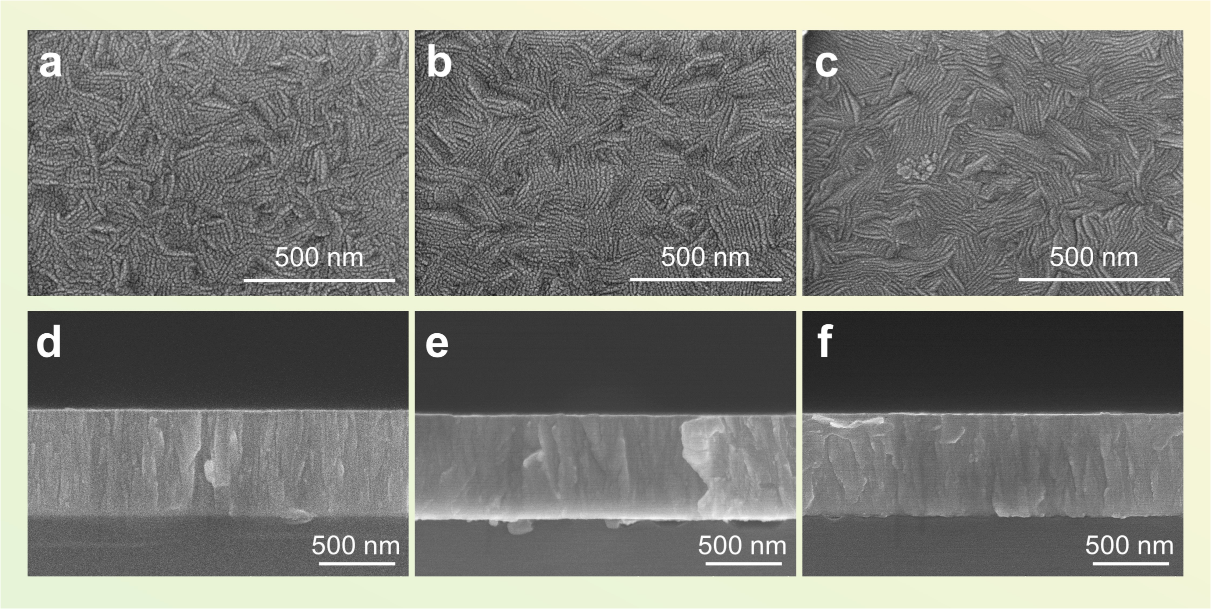

Supplementary Fig. 7 The SEM images of Nb-Mo-Ta-W high entropy alloy films with 600 nm. **a, d** $Nb_{25}Mo_{25}Ta_{25}W_{25}$. **b, e** $Nb_{15}Mo_{15}Ta_{35}W_{35}$. **c, f** $Nb_{15}Mo_{35}Ta_{15}W_{35}$.

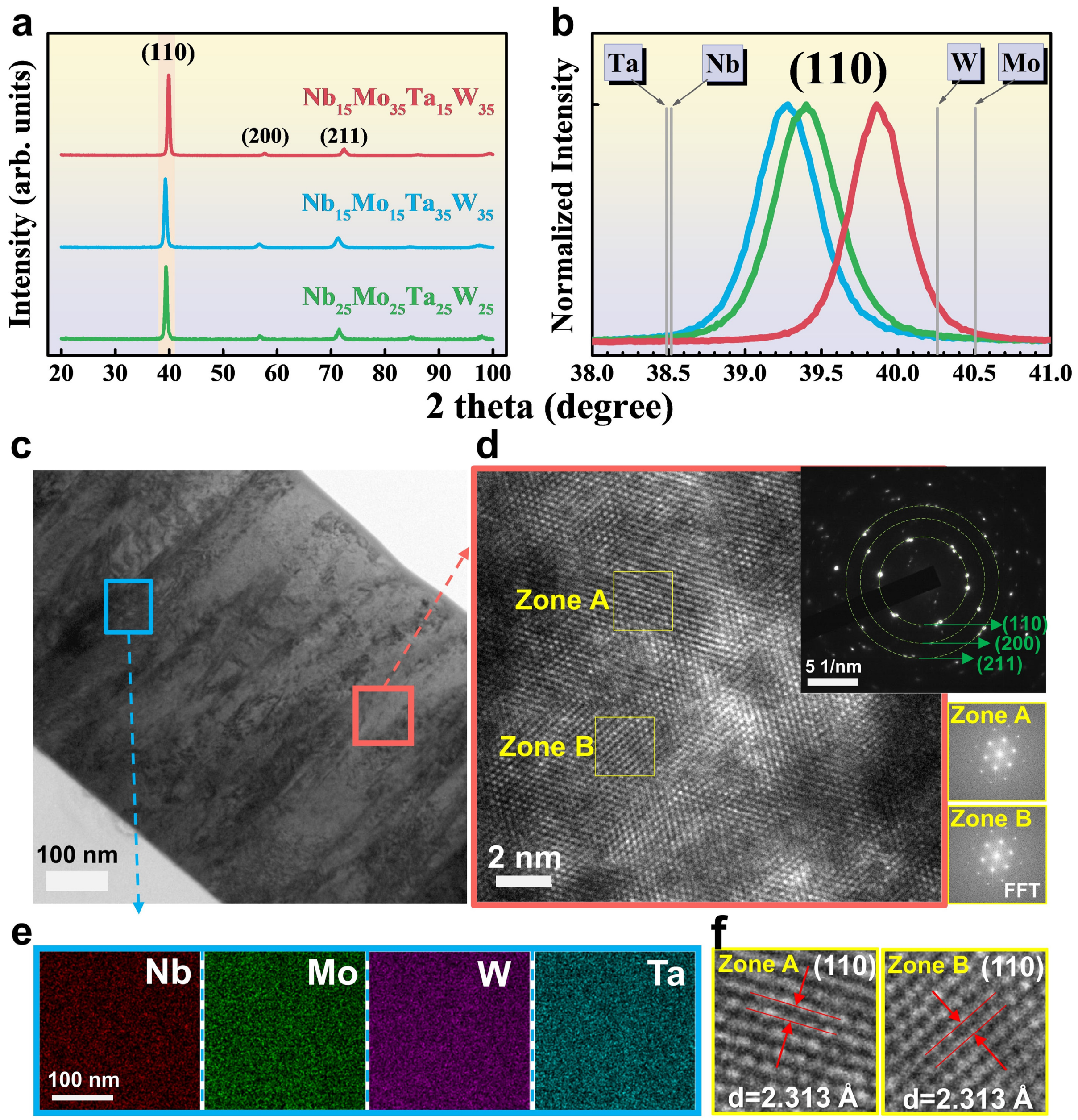

Supplementary Fig. 8 Solid solution and crystalline characteristics analyses. a, b XRD pattern of three NbMoTaW films. **c, d** HRTEM images. **e** EDS mapping of the equal molar NbMoTaW film. **f** Inverse Fourier analysis of the plane distance (**d**) of NbMoTaW. The value of d~2.313 Å corresponds to BCC (110).

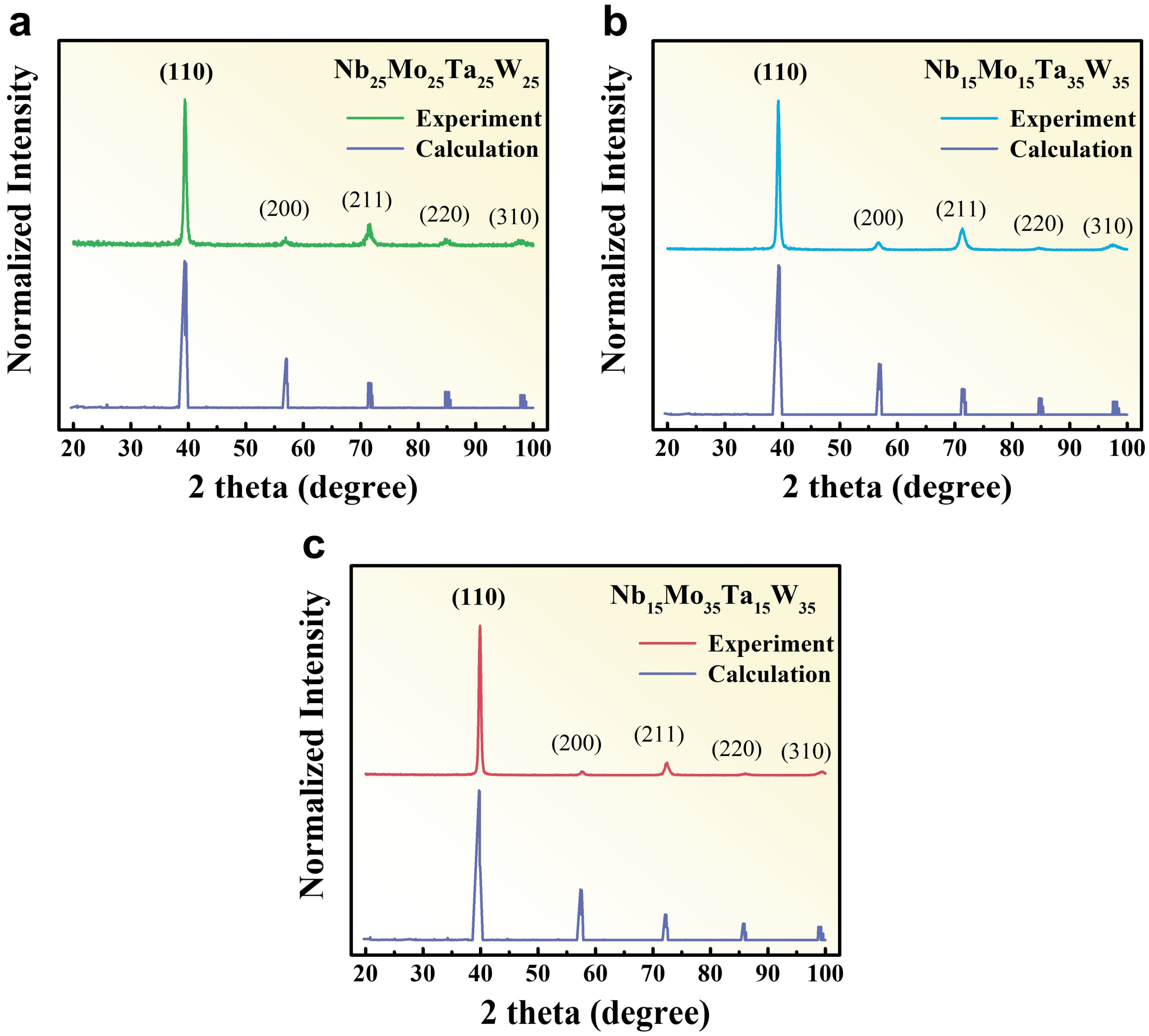

Supplementary Fig. 9 Experimental and calculation results of XRD pattern of NbMoTaW films. Consistent results are obtained. **a** $Nb_{25}Mo_{25}Ta_{25}W_{25}$. **b** $Nb_{15}Mo_{15}Ta_{35}W_{35}$. **c** $Nb_{15}Mo_{35}Ta_{15}W_{35}$.

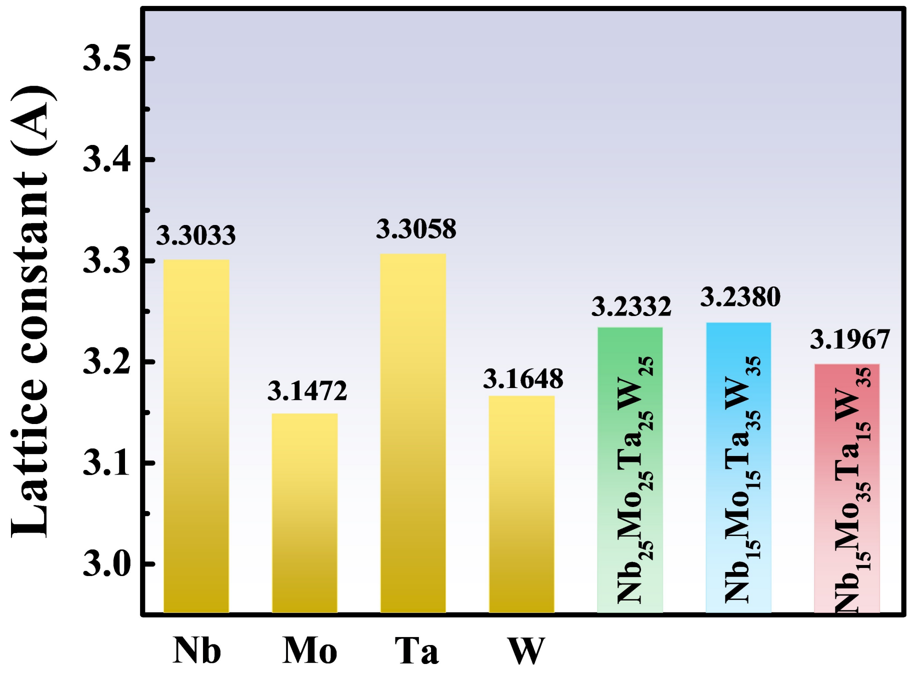

Supplementary Fig. 10 Lattice constant of three NbMoTaW films obtained from XRD. The lattice constants of Nb, Mo, Ta, and W plotted for reference.

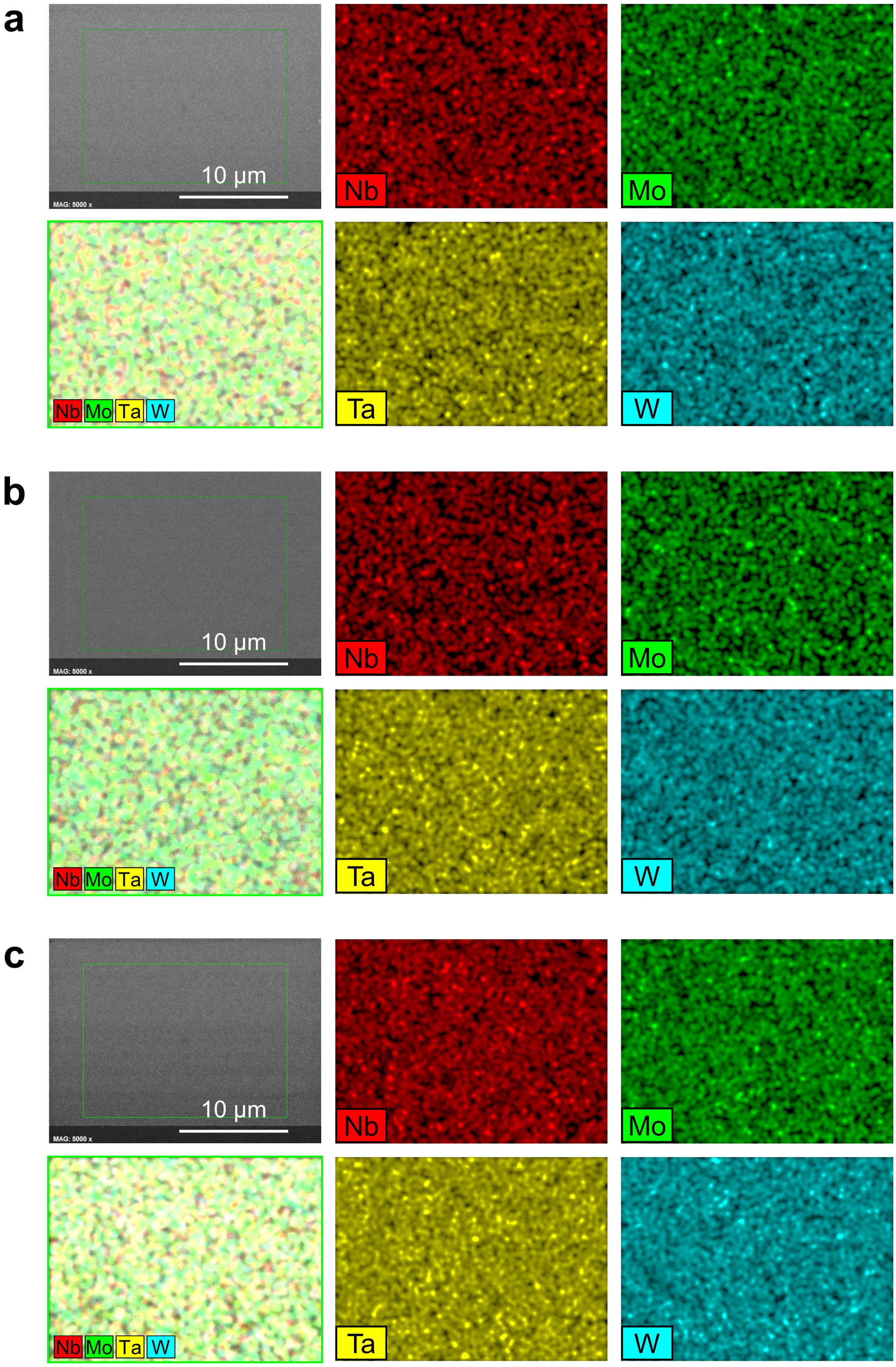

Supplementary Fig. 11 EDS elemental mapping micrographs of the NbMoTaW films. **a** $Nb_{25}Mo_{25}Ta_{25}W_{25}$. **b** $Nb_{15}Mo_{15}Ta_{35}W_{35}$. **c** $Nb_{15}Mo_{35}Ta_{15}W_{35}$.

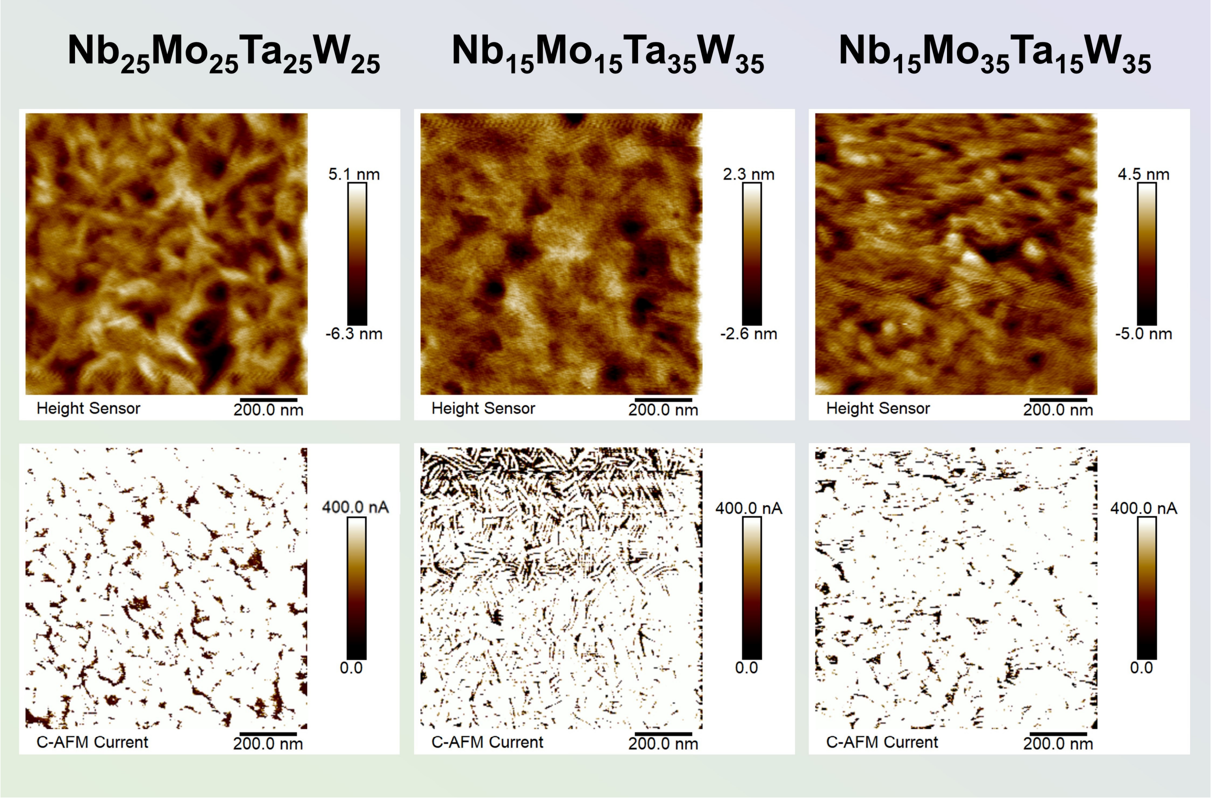

Supplementary Fig. 12 Morphology(upper figures) and current mapping(lower figures) by CAFM of HEA film with 1200 nm. $Nb_{15}Mo_{35}Ta_{15}W_{35}$ shows the most high-level current among all, indicating it has the best conductivity.

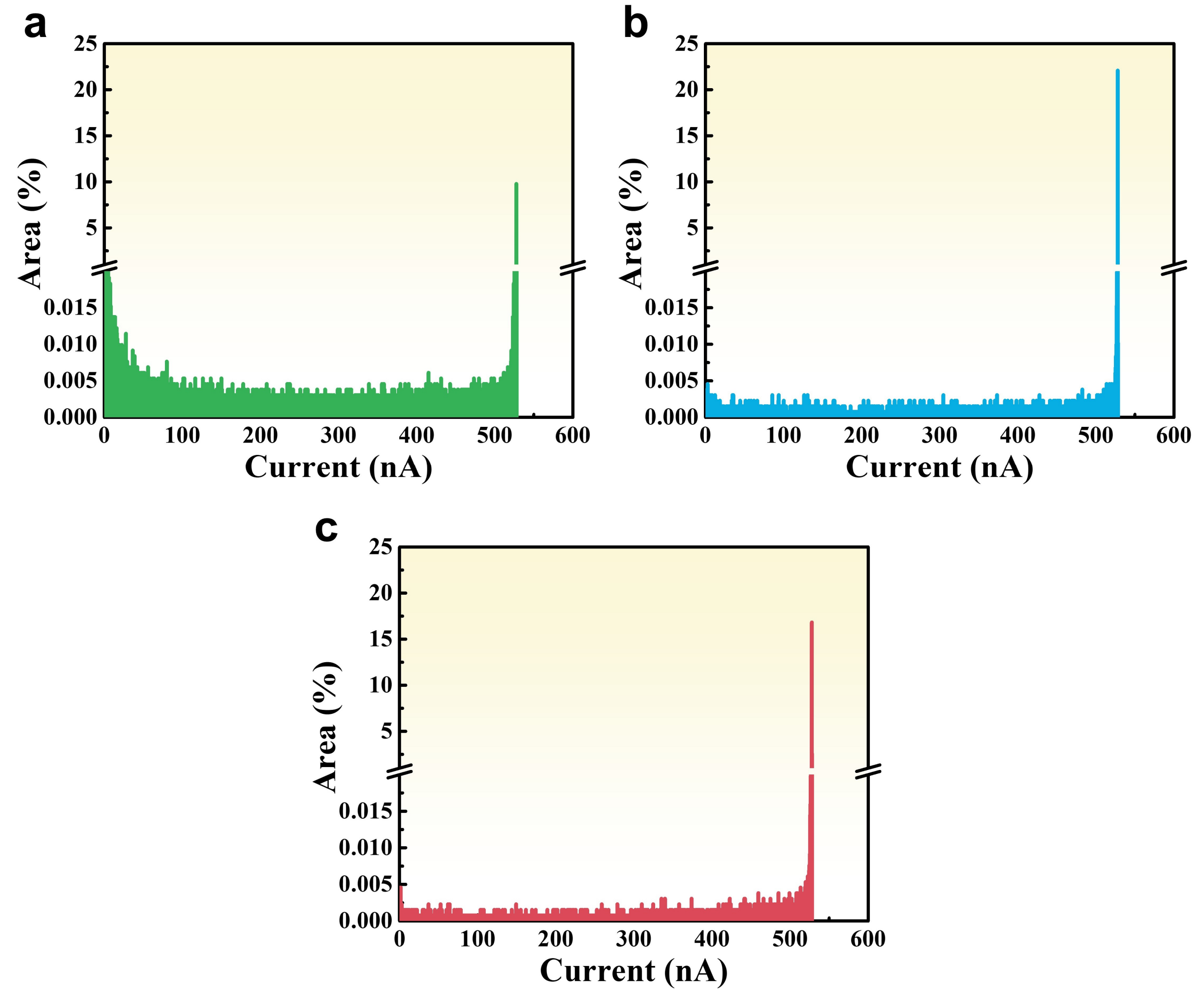

Supplementary Fig. 13 Conductive area of current mapping by CAFM of HEA films with 1200 nm. **a** $Nb_{25}Mo_{25}Ta_{25}W_{25}$. **b** $Nb_{15}Mo_{15}Ta_{35}W_{35}$. **c** $Nb_{15}Mo_{35}Ta_{15}W_{35}$. The integration of the conducting area is 96.1%, 95.5%, and 98.9%, respectively.

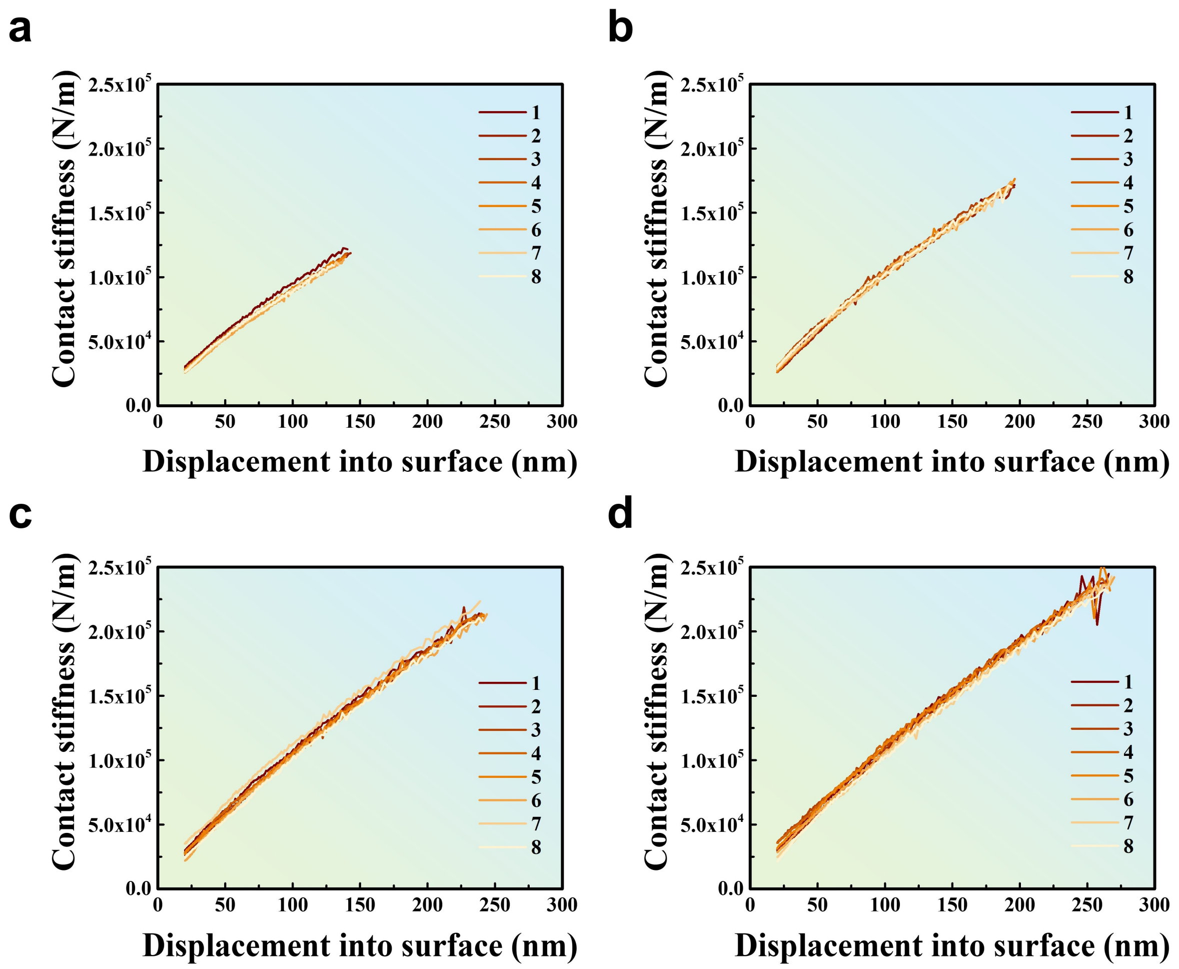

Supplementary Fig. 14 The continuous stiffness measurement method was used to measure the relationship between the modulus of different thicknesses of $Nb_{25}Mo_{25}Ta_{25}W_{25}$ films and the depth of indentation. **a** 300 nm. **b** 600 nm. **c** 900 nm. **d** 1200 nm. (Number of samples: 1-8)

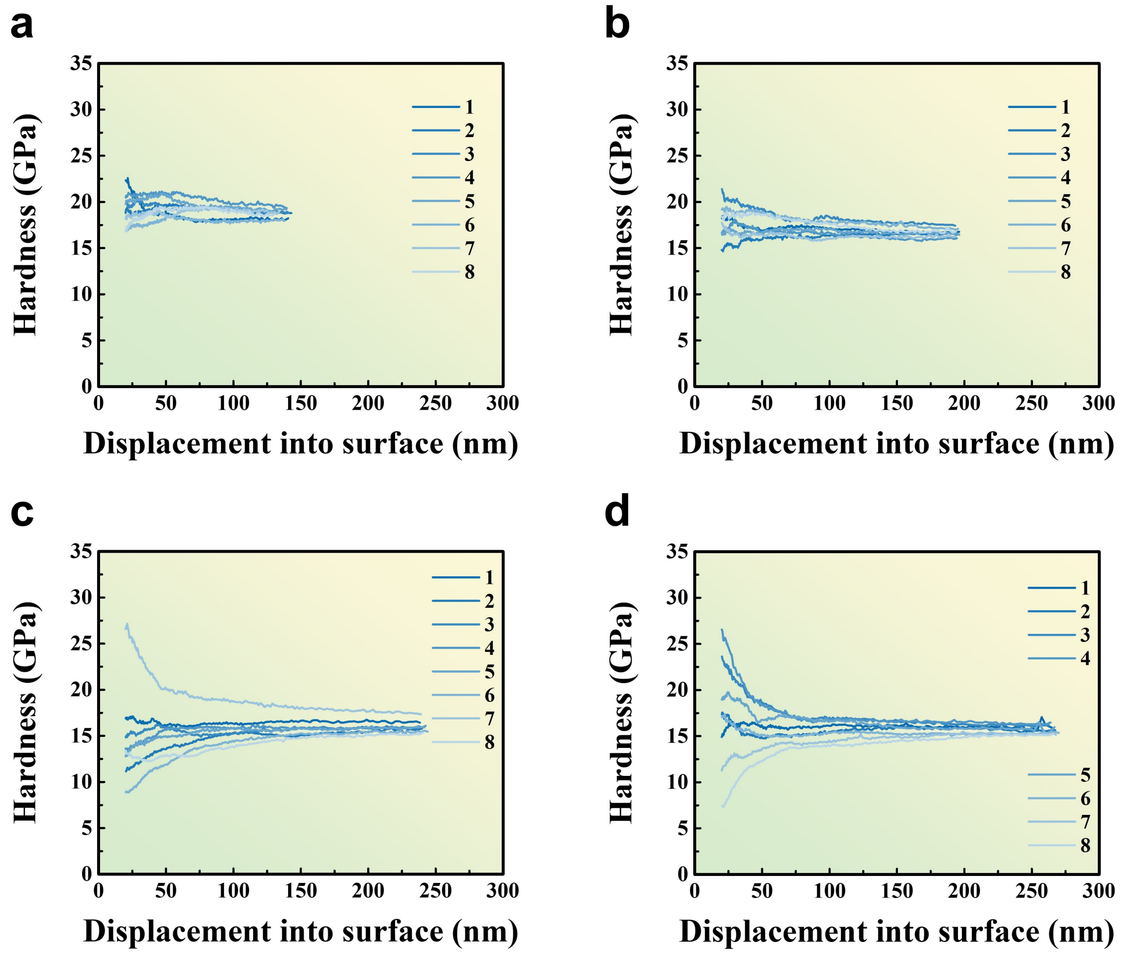

Supplementary Fig. 15 The continuous stiffness method was employed to measure the relationship between the hardness and indentation depth of $Nb_{25}Mo_{25}Ta_{25}W_{25}$ films with varying thickness. **a** 300 nm. **b** 600 nm. **c** 900 nm. **d** 1200 nm. (Number of samples: 1-8)

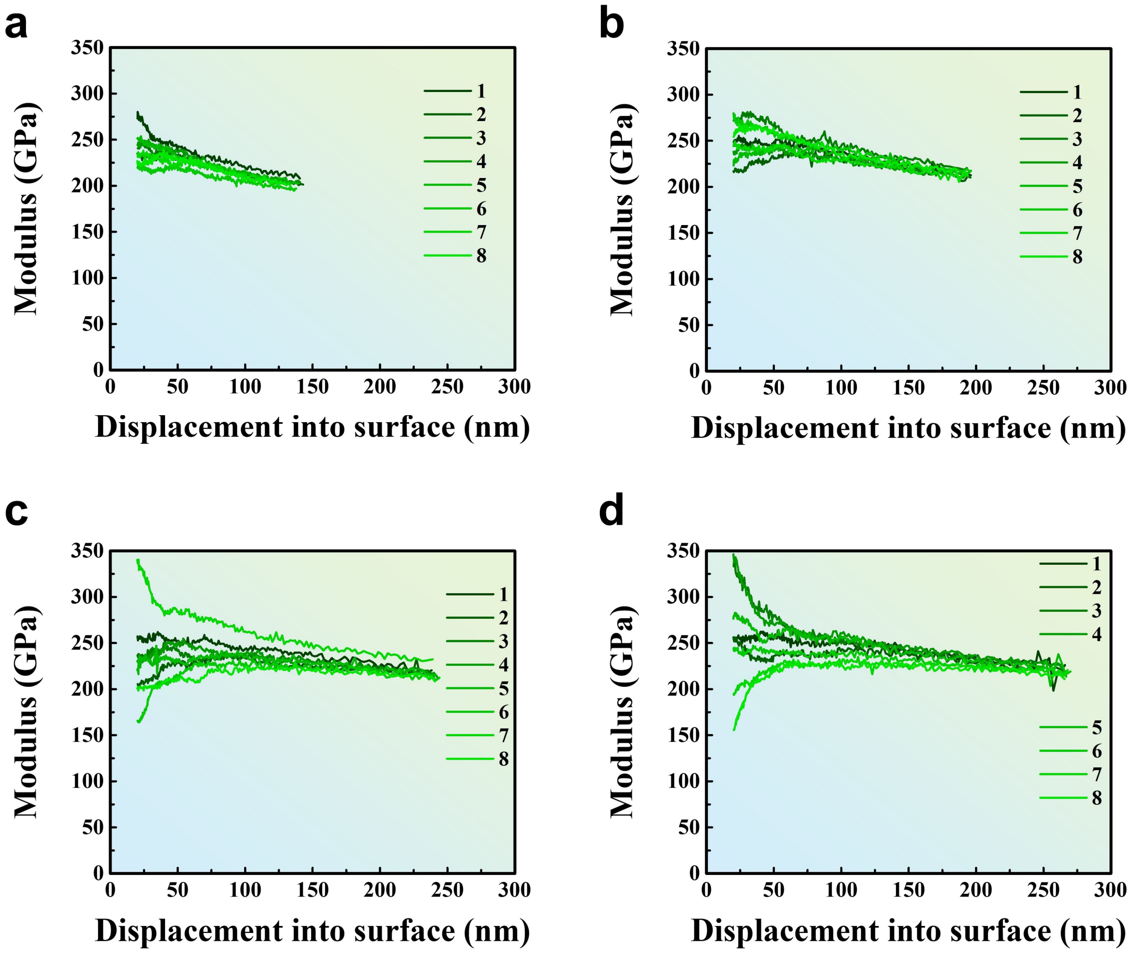

Supplementary Fig. 16 The continuous stiffness method was employed to measure the relationship between the Young's modulus and indentation depth of $Nb_{25}Mo_{25}Ta_{25}W_{25}$ films with varying thickness. **a** 300 nm. **b** 600 nm. **c** 900 nm. **d** 1200 nm. (Number of samples: 1-8)

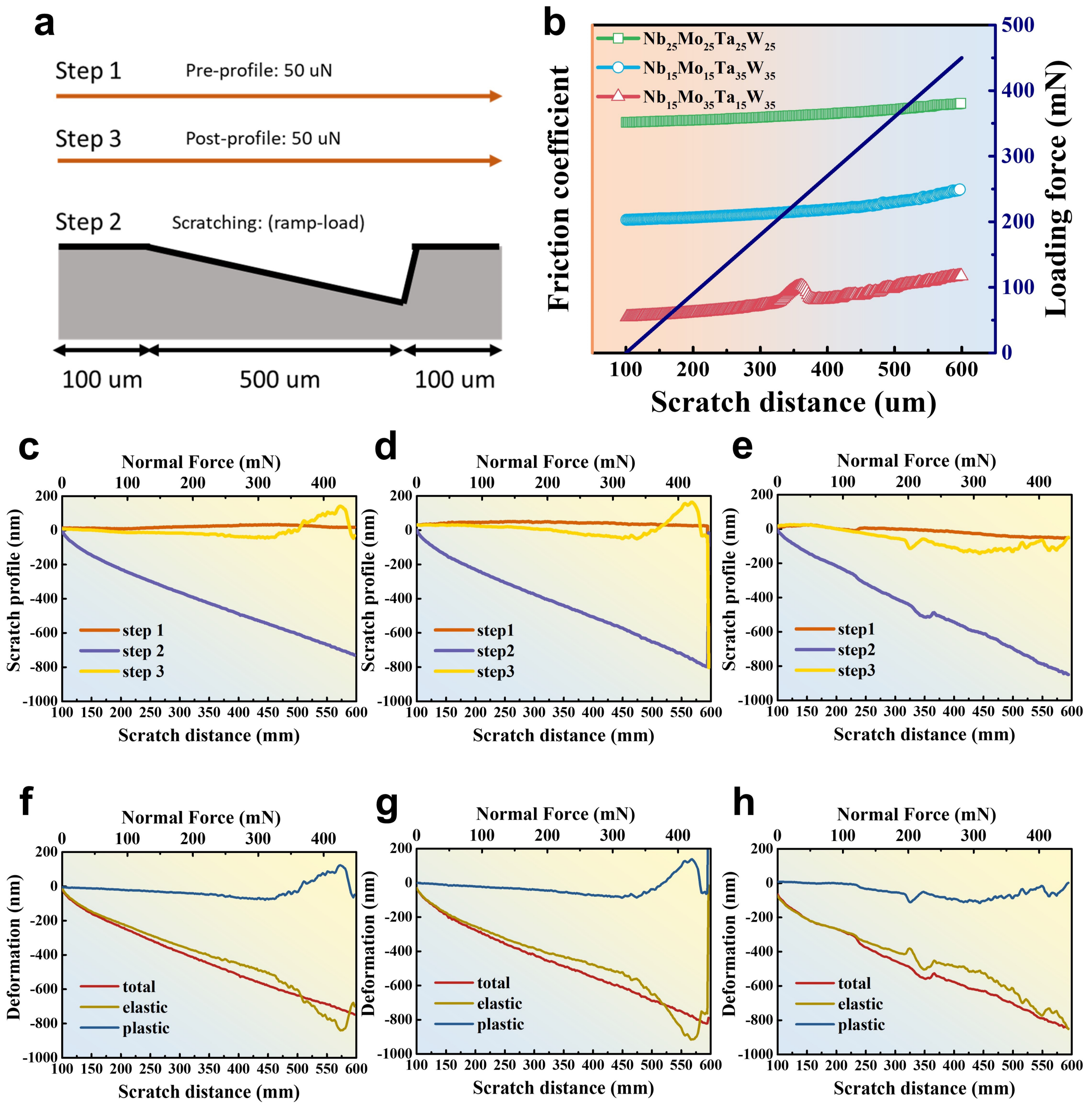

Supplementary Fig. 17 Results of scratching test. a Schematic of the three steps in nano-scratch testing. **b** Friction coefficient and loading force with scratch distance. The surface morphology changes of the film during nano-scratch testing process of **c** $Nb_{25}Mo_{25}Ta_{25}W_{25}$, **d** $Nb_{15}Mo_{15}Ta_{35}W_{35}$, and **e** $Nb_{15}Mo_{35}Ta_{15}W_{35}$. The elasticity, plasticity, and total deformation after nano-scratch test of **f** $Nb_{25}Mo_{25}Ta_{25}W_{25}$, **g** $Nb_{15}Mo_{15}Ta_{35}W_{35}$, and **h** $Nb_{15}Mo_{35}Ta_{15}W_{35}$.

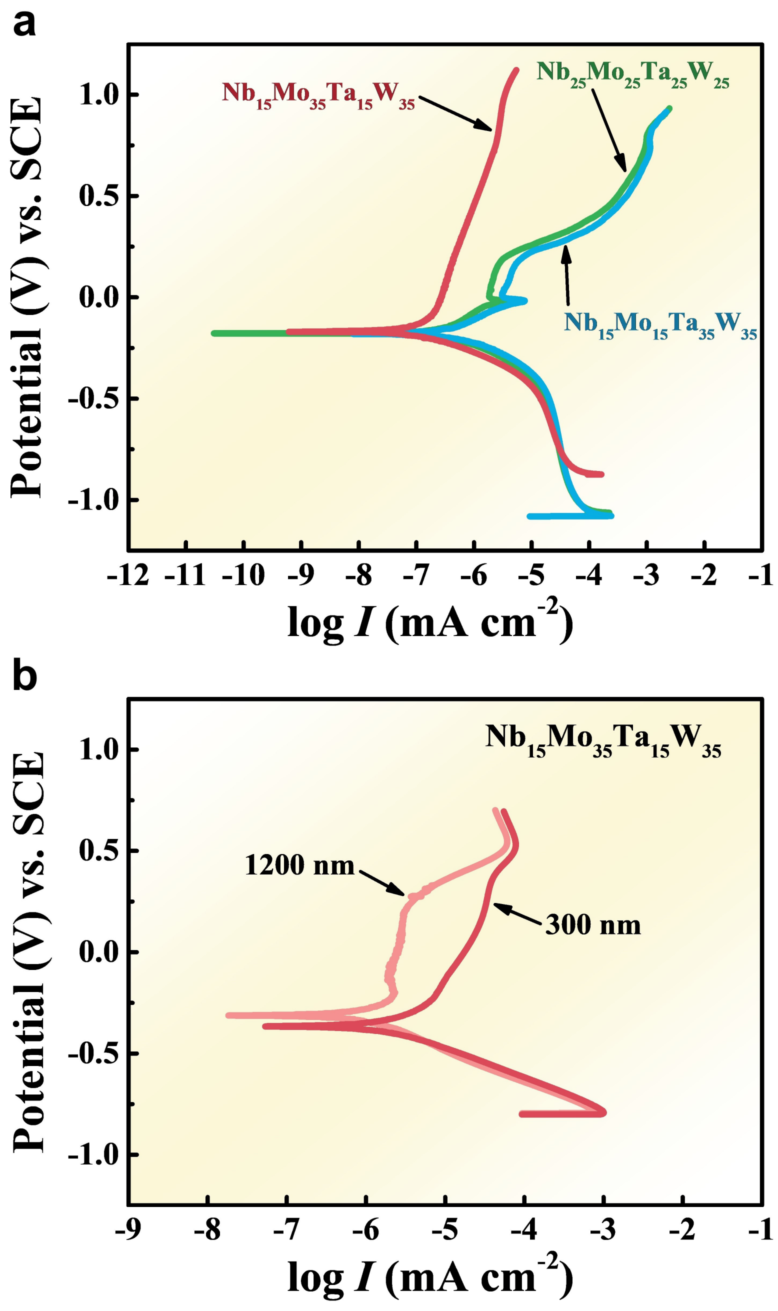

Supplementary Fig. 18 Potentiodynamic polarization curves. a $Nb_{25}Mo_{25}Ta_{25}W_{25}$, $Nb_{15}Mo_{15}Ta_{35}W_{35}$, and $Nb_{15}Mo_{35}Ta_{15}W_{35}$ films with 300 nm in 3.5wt.% NaCl solution. **b** $Nb_{15}Mo_{35}Ta_{15}W_{35}$ films with 300 and 1200 nm in 0.1M H_2SO_4 solution.

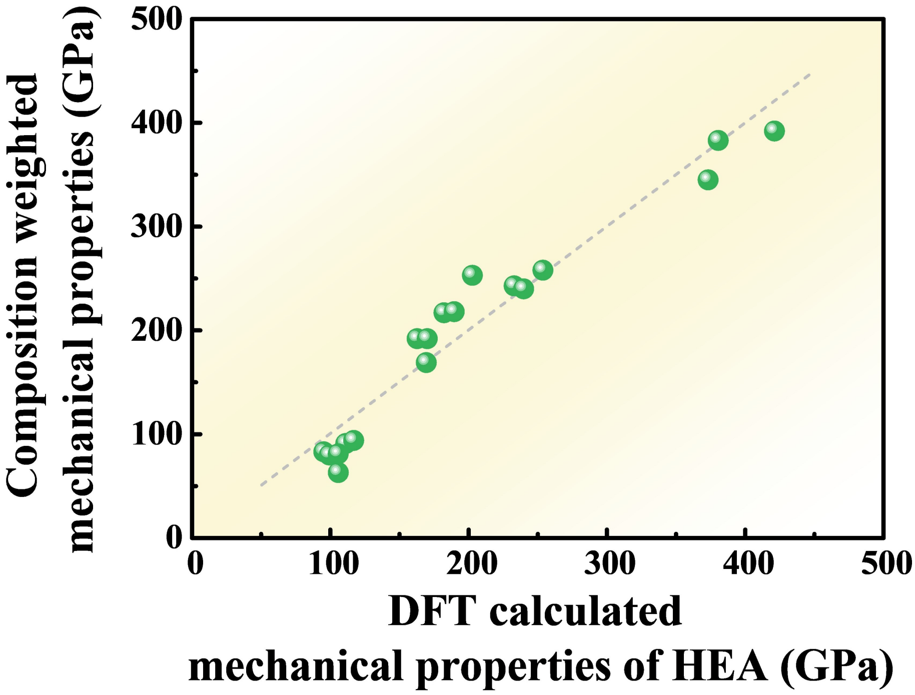

Supplementary Fig. 19 Compositional weighted mechanical properties versus DFT calculated mechanical properties of the NbMoTaW HEA system in this work. Mechanical properties include C_{11}, C_{12}, C_{44}, Bulk modulus, Shear modulus and Young's modulus.

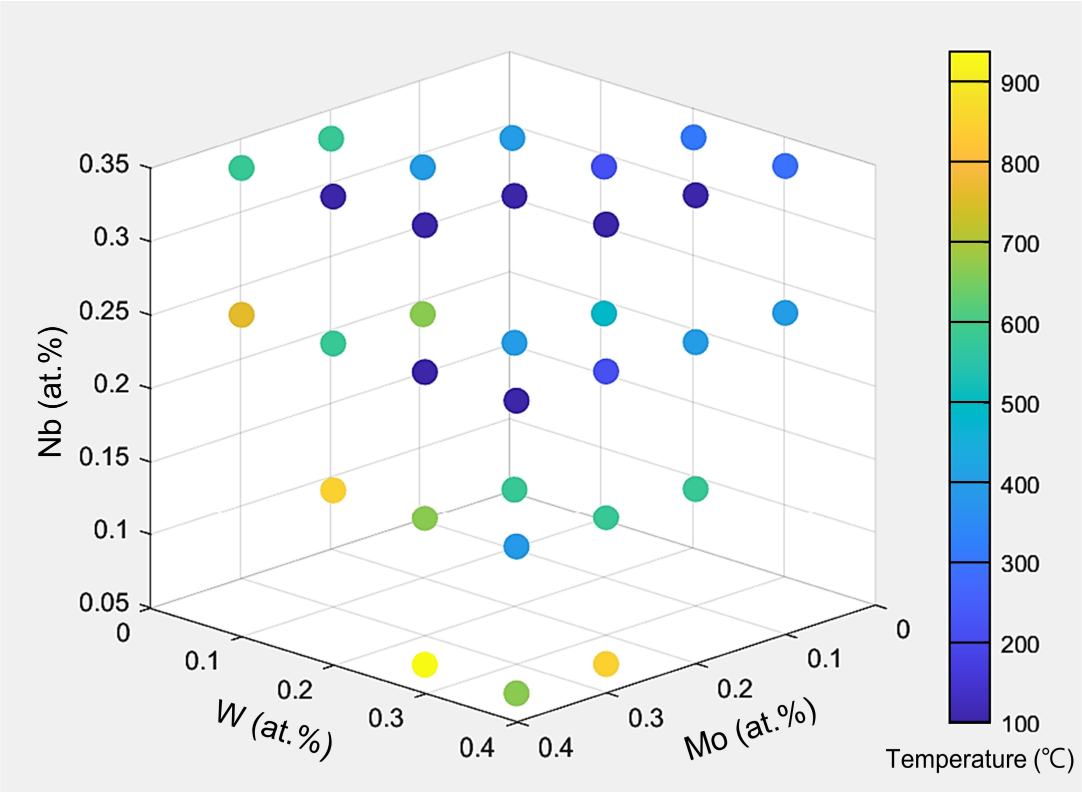

Supplementary Fig. 20 The transformation temperatures (℃) from the HEA phase to the B2 phase are plotted based on the composition ratios of Nb, Mo, and W.

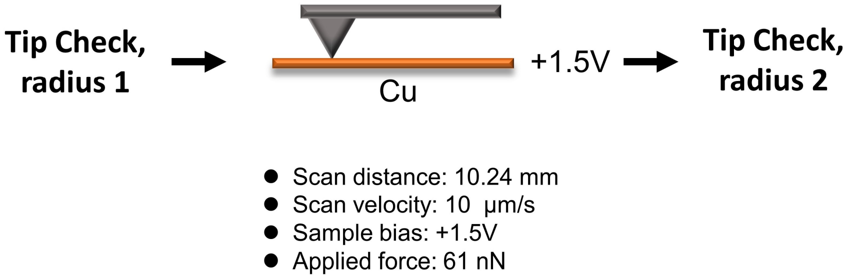

Supplementary Fig. 21 Method of wearing test on AFM probe. The wear test in this study utilized a PPP-NCSTR-50 probe. Prior to conducting the wear test, Blind Tip Reconstruction (BTR) was employed to obtain the three-dimensional morphology of the probe before wear. The probe scanned the surface of the copper adhesive with a force of 61 nN, covering a total scanning length of 10.24 mm, while applying a bias voltage of 1.5 V to perform the wear test. Finally, BTR was used again to obtain the three-dimensional morphology of the probe after wear. The wear resistance was assessed by calculating the rate of change in the tip radius of the probe before and after wear.

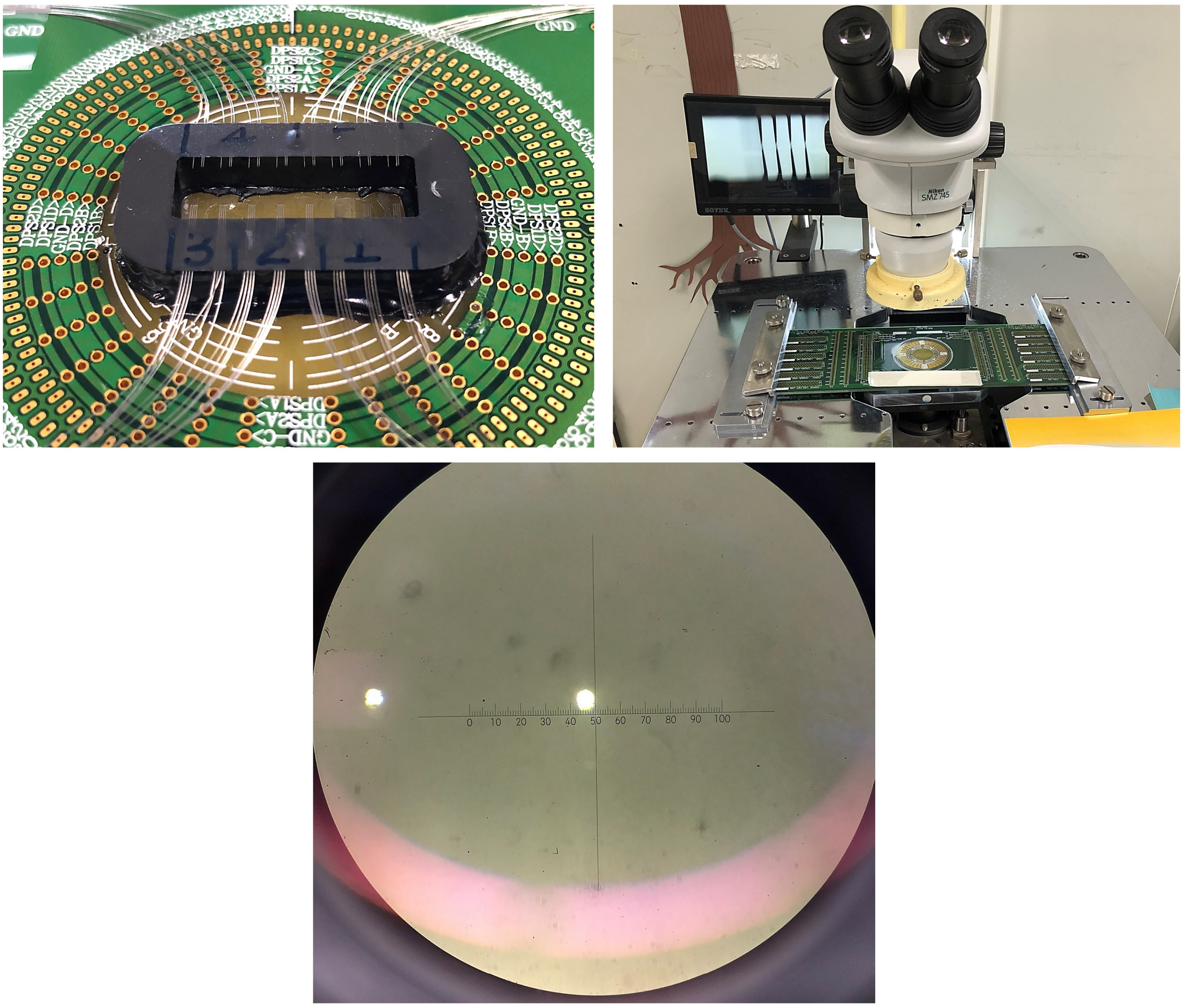

Supplementary Fig. 22 Schematic illustration of the wear test of semiconductor chip testing probe - ReW tip, undergoing 200,000 clicks.

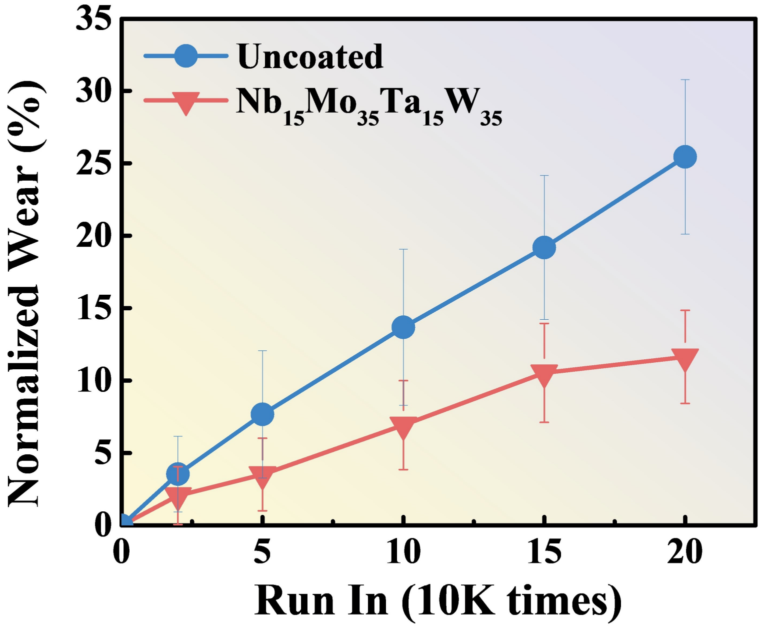

Supplementary Fig. 23 Wearing test results of the ReW probe tipped with and without $Nb_{15}Mo_{35}Ta_{15}W_{35}$ high-entropy alloy film after 200,000 clicks. The error bars represent the standard deviation.

Supplementary Table 1 The experimental values of conductivity, plasma frequency, and relaxation time for Nb, Mo, Ta, and W indicate that Mo and W have the highest conductivity, followed by Ta, while Nb has the lowest conductivity[10].

Element	Conductivity ($S\ m^{-1}$)_Exp. at 20°C	ω_{Dp} (eV)	Relaxation time (s)
Nb	6.90×10^{6}	9.11	4.07×10^{-15}
Mo	1.89×10^{7}	9.01	11.4×10^{-15}
Ta	7.60×10^{6}	8.86	4.73×10^{-15}
W	1.89×10^{7}	7.93	14.7×10^{-15}

Supplementary Table 2 The relaxation time and DC conductivity of three different compositions of NbMoTaW high entropy alloys.

High entropy alloy		ω_{Dp} (eV)	Relaxation Time (s)	DC conductivity (S m^{-1})
$Nb_{25}Mo_{25}Ta_{25}W_{25}$	Exp.	-	-	1.39×10^{6}
	Cal. Method 1	-	-	1.31×10^{7}
	Cal. Method 2	4.54	8.73×10^{-15}	3.67×10^{6}
	Cal. Method 3		1.99×10^{-15}	0.84×10^{6}
$Nb_{15}Mo_{15}Ta_{35}W_{35}$	Exp.	-	-	0.98×10^{6}
	Cal. Method 1	-	-	1.31×10^{7}
	Cal. Method 2	3.77	9.12×10^{-15}	2.66×10^{6}
	Cal. Method 3		2.04×10^{-15}	0.59×10^{6}
$Nb_{15}Mo_{35}Ta_{15}W_{35}$	Exp.	-	-	2.51×10^{6}
	Cal. Method 1	-	-	1.54×10^{7}
	Cal. Method 2	4.66	10.5×10^{-15}	4.65×10^{6}
	Cal. Method 3		2.00×10^{-15}	0.89×10^{6}

Supplementary Table 3 The plasma frequency, free electron density, and effective electron mass of the elements Nb, Mo, Ta, W, and the high entropy alloys $Nb_{25}Mo_{25}Ta_{25}W_{25}$, $Nb_{15}Mo_{15}Ta_{35}W_{35}$, $Nb_{15}Mo_{35}Ta_{15}W_{35}$.

Materials	ω_{Dp} (eV)	Free electron density (electron m^{-3})	Effective mass (electron mass)
Nb	9.11	3.28×10^{28}	1.01
Mo	9.01	1.71×10^{28}	2.86
Ta	8.86	1.39×10^{28}	1.41
W	7.93	1.01×10^{28}	0.85
$Nb_{25}Mo_{25}Ta_{25}W_{25}$	4.54	2.56×10^{26}	6.26
$Nb_{15}Mo_{15}Ta_{35}W_{35}$	3.77	3.21×10^{26}	4.61
$Nb_{15}Mo_{35}Ta_{15}W_{35}$	4.66	2.47×10^{26}	5.79

Supplementary Table 4 The relaxation time, lattice constant, and Mean Square Displacement (MSD) of the elements Nb, Mo, Ta, W, and the high entropy alloys $Nb_{25}Mo_{25}Ta_{25}W_{25}$, $Nb_{15}Mo_{15}Ta_{35}W_{35}$, $Nb_{15}Mo_{35}Ta_{15}W_{35}$.

Element	Relaxation time (s)	Lattice constant (Å)	Mean square displacement of atoms (Å)
Nb	4.07×10^{-15}	3.3	0
Mo	11.4×10^{-15}	3.14	0
Ta	4.73×10^{-15}	3.3	0
W	14.7×10^{-15}	3.16	0
**High entropy alloy**	**Relaxation time (s)**	**Deviation of Lattice constant (%)**	**Mean square displacement of atoms (Å)**
$Nb_{25}Mo_{25}Ta_{25}W_{25}$	1.99×10^{-15}	0.04%	7.25×10^{-8}
$Nb_{15}Mo_{15}Ta_{35}W_{35}$	2.04×10^{-15}	0.14%	4.29×10^{-3}
$Nb_{15}Mo_{35}Ta_{15}W_{35}$	2.00×10^{-15}	-0.72%	6.91×10^{-8}

Supplementary Table 5 The calculation results of the derailed lattice data of NbMoTaW thin films.

$Nb_{25}Mo_{25}Ta_{25}W_{25}$	*a*	*b*	*c*	*α*	*β*	*γ*
model1	3.23	3.23	3.22	90.15	90.31	90.11
model2	3.23	3.22	3.23	89.63	90.15	90.09
model3	3.22	3.23	3.23	90.27	90.01	90.06
average	3.23	3.22	3.23	90.02	90.16	90.09
$Nb_{15}Mo_{15}Ta_{35}W_{35}$	*a*	*b*	*c*	*α*	*β*	*γ*
model1	3.23	3.23	3.23	90.04	89.94	89.68
model2	3.22	3.23	3.24	90.06	90.00	90.05
model3	3.23	3.22	3.24	90.20	89.92	90.10
average	3.23	3.23	3.24	90.10	89.95	89.94
$Nb_{15}Mo_{35}Ta_{15}W_{35}$	*a*	*b*	*c*	*α*	*β*	*γ*
model1	3.20	3.20	3.21	90.00	90.02	90.16
model2	3.20	3.20	3.21	89.96	90.04	89.97
model3	3.20	3.20	3.21	90.04	90.02	89.92
average	3.20	3.20	3.21	90.00	90.03	90.01

Supplementary Table 6 Experimental results of the crystalline properties of the NbMoTaW films obtained from XRD.

High entropy alloy	Lattice constant (Å)	*2θ*	*d* (Å)	FWHM	Grain size (nm)
$Nb_{25}Mo_{25}Ta_{25}W_{25}$	3.2332	39.39°	2.2854	0.491°	16.95
$Nb_{15}Mo_{15}Ta_{35}W_{35}$	3.2380	39.28°	2.2920	0.475°	15.88
$Nb_{15}Mo_{35}Ta_{15}W_{35}$	3.1967	39.87°	2.2593	0.423°	19.98

Supplementary Table 7 Current mapping by CAFM of NbMoTaW films with 1200 nm.

High entropy alloy	Area (%)		Effective current (μA)
$Nb_{25}Mo_{25}Ta_{25}W_{25}$	Dark	3.87	21.52
	Bright	96.13	
$Nb_{15}Mo_{15}Ta_{35}W_{35}$	Dark	4.51	18.32
	Bright	95.49	
$Nb_{15}Mo_{35}Ta_{15}W_{35}$	Dark	1.11	25.39
	Bright	98.89	

Supplementary Table 8 Corrosion parameters obtained from potentiodynamic polarization curves of NbMoTaW films and common alloy materials in 3.5 wt.% NaCl solution and 0.1 M H_2SO_4 solution. (Note: The 3.5 wt.% NaCl solution is approximately the condition of seawater.)

Materials	Thickness (nm)	Solution	i_{corr} (μA cm^{-2})	E_{corr} (V_{SCE})
$Nb_{25}Mo_{25}Ta_{25}W_{25}$	300	3.5 wt.% NaCl	0.87	-0.178
$Nb_{15}Mo_{15}Ta_{35}W_{35}$	300	3.5 wt.% NaCl	0.78	-0.180
$Nb_{15}Mo_{35}Ta_{15}W_{35}$	300	3.5 wt.% NaCl	0.76	-0.168
$Nb_{15}Mo_{35}Ta_{15}W_{35}$	300	0.1 M H_2SO_4	2.81	-0.367
$Nb_{15}Mo_{35}Ta_{15}W_{35}$	1200	0.1 M H_2SO_4	2.91	-0.316
AISI 304 [11]	-	3.5 wt.% NaCl	7	-0.482
γ-TiAl [12]	-	3.5 wt.% NaCl	1.94	-0.428
SS316L [13]	-	Seawater	0.26	-0.274

Supplementary Table 9 Mechanical properties [14], elastic constants C_{11}, C_{12}, C_{44} (GPa), bulk modulus B (GPa), shear modulus G (GPa), Young's modulus E (GPa), and hardness (GPa).

(Gpa)	C_{11}	C_{12}	C_{44}	B	G	E	Hardness
$Nb_{25}Mo_{25}Ta_{25}W_{25}$	345	192	83	243	80	217	6.68
$Nb_{15}Mo_{15}Ta_{35}W_{35}$	383	169	63	240	81	218	6.88
$Nb_{15}Mo_{35}Ta_{15}W_{35}$	392	192	91	258	94	253	8.30
NbMoTaW (DFT) [14]	414	186	69	262	84	229	6.66
NbMoTaW (DFT) [14]	371	160	69	230	82	223	7.44

Supplementary References

1 Gallagher, P. *et al.* Quantum-critical conductivity of the Dirac fluid in graphene. *Science* **364**, 158-162 (2019).

2 Barcons Ruiz, D. *et al.* Experimental signatures of the transition from acoustic plasmon to electronic sound in graphene. *Science Advances* **9**, eadi0415 (2023).

3 Samizadeh Nikoo, M. & Matioli, E. Electronic metadevices for terahertz applications. *Nature* **614**, 451-455 (2023).

4 Wu, P.-J., Hung, J.-T., Hsieh, C.-F., Yang, C.-R. & Yang, C.-S. High-selectivity terahertz metamaterial nitric oxide sensor based on ZnTiO3 perovskite membrane. *APL Photonics* **8** (2023).

5 Peng, K. *et al.* Three-dimensional cross-nanowire networks recover full terahertz state. *Science* **368**, 510-513 (2020).

6 Kutas, M. *et al.* Terahertz quantum sensing. *Science Advances* **6**, eaaz8065 (2020).

7 Chen, S. L. *et al.* The PANDAT software package and its applications. *Calphad* **26**, 175-188 (2002).

8 Yeh, C.-H. *et al.* Design of high-entropy films as ultra-violet light reflector. *Applied Materials Today* **36**, 102013 (2024).

9 Han, K., Jiang, H., Huang, T. & Wei, M. Thermoelectric Properties of CoCrFeNiNbx Eutectic High Entropy Alloys. *Crystals* **10**, 762 (2020).

10 Lide, D. R. *CRC Handbook of Chemistry and Physics*. 80th edn, (CRC Press, 1999).

11 Zhang, G. *et al.* Microstructure and Properties of AlCoCrFeNiSi High-Entropy Alloy Coating on AISI 304 Stainless Steel by Laser Cladding. *Journal of Materials Engineering and Performance* **29**, 278-288 (2020).

12 Delgado-Alvarado, C. & Sundaram, P. A. A study of the corrosion behavior of gamma titanium aluminide in 3.5wt% NaCl solution and seawater. *Corrosion Science* **49**, 3732-3741 (2007).

13 Meghwal, A. *et al.* Multiscale mechanical performance and corrosion behaviour of plasma sprayed AlCoCrFeNi high-entropy alloy coatings. *Journal of Alloys and Compounds* **854**, 157140 (2021).

14 Fazakas, É. *et al.* Experimental and theoretical study of Ti20Zr20Hf20Nb20X20 (X=V or Cr) refractory high-entropy alloys. *International Journal of Refractory Metals and Hard Materials* **47**, 131-138 (2014).
